# Granulomatous lymphadenitis in the inferior pulmonary ligament

**DOI:** 10.1259/bjrcr.20220087

**Published:** 2022-09-26

**Authors:** Francis Girvin, Fang Zhou, Joanna Escalon, Sharon Steinberger, Deepti Gupta, Lauren Groner

**Affiliations:** 1 Department of Radiology, Presbyterian / Weill Cornell Medical Center, NewYork, United States; 2 Department of Pathology, University Langone Health, New York, United States

## Abstract

The inferior pulmonary ligament and related connective tissue septa are a recognizable site of granulomatous lymphadenitis on CT of the chest and may mimic a lung parenchymal lesion. The anatomy of the inferior pulmonary ligament, CT appearances and potential etiologies of this entity are reviewed and illustrated.

## Introduction

Although inflammatory granulomatous conditions affecting the lung are well-described, the occurrence of granulomatous lymphadenitis within the inferior pulmonary ligament has not been recognized as a distinct entity to date. An awareness of the anatomy and normal appearance of the inferior pulmonary ligament (IPL) facilitates recognition of a lobulated nodular density at this site as a potential case of granulomatous lymphadenitis (GL), and to distinguish this condition from a parenchymal-based lesion.

## Discussion

**Figure 1.**
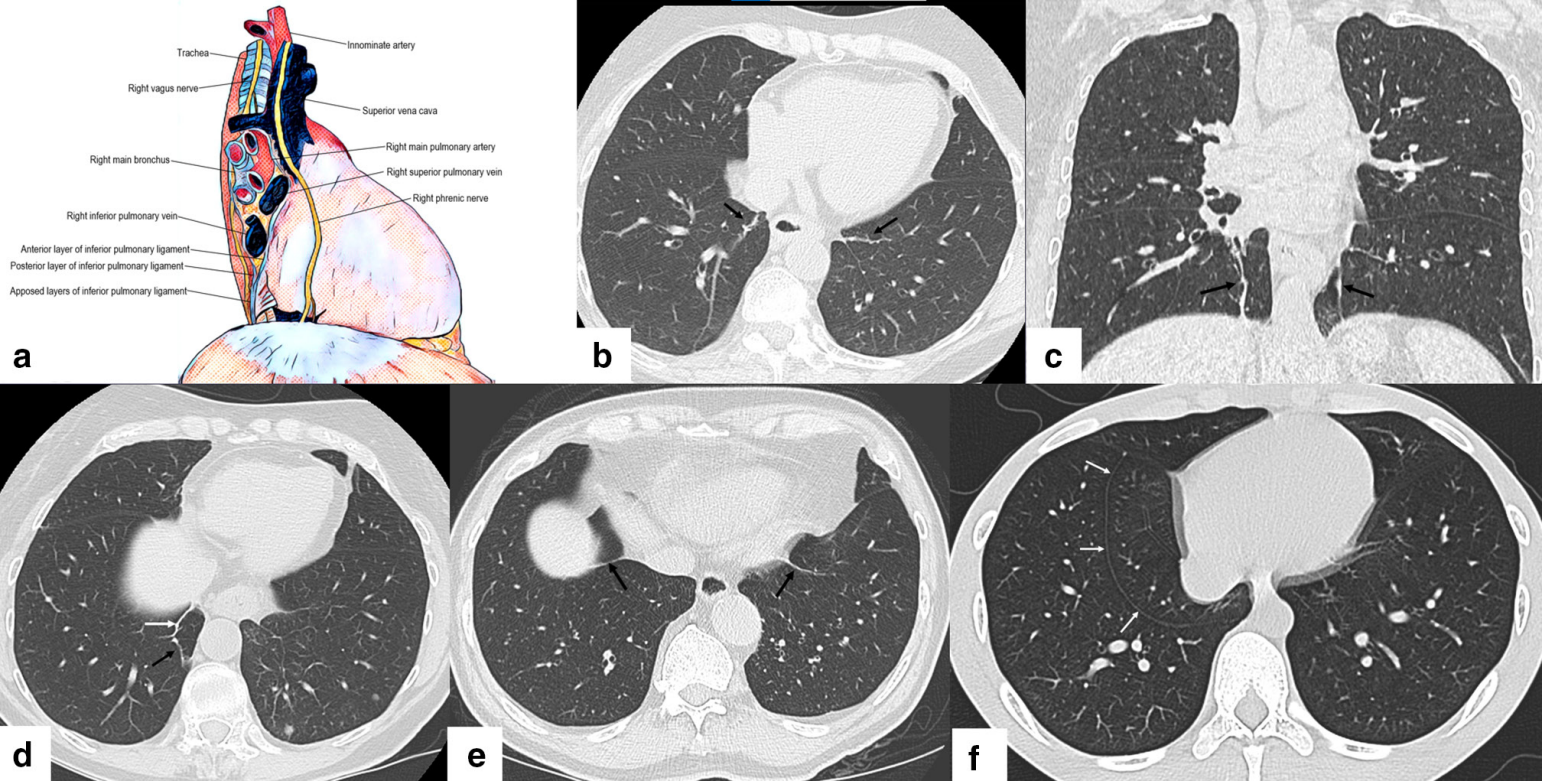
A. Labelled medical illustration of the anatomy of the inferior pulmonary ligament Figure 1B. Axial chest CT image on lung windows showing left and right IPLs (black arrows) Figure 1C. Coronal oblique chest CT image on lung windows showing left and right IPLs (black arrows) Figure 1D. Axial chest CT image on lung windows showing an intersegmental septum (black arrow) contiguous with the IPL (white arrow) Figure 1E. Axial chest CT image on lung windows showing bilateral pericardiophrenic bundles (black arrows) Figure 1F. Axial chest CT image on lung windows showing inferior accessory fissure in right lower lobe (white arrows). IPL, inferior pulmonary ligament

The pleura comprises two serosal membranes—an inner visceral layer that covers the lung, and an outer parietal layer covering the internal chest wall. The transition between visceral and parietal pleura occurs at the lung hilum, where the pleura surrounds the upper, anterior and posterior hilar structures.^1–3^ Below the hilum, the anterior and posterior pleural layers become apposed to form the inferior pulmonary (or “triangular”) ligament (Figure 1A), containing lose connective tissue, small bronchial and esophageal arterial branch vessels, small venous tributaries of the superior diaphragmatic veins, and lymphatics and nodes draining the lower lobes and distal esophagus.^1,2^ The IPL is enveloped both anteriorly and posteriorly by lung and serves as an anchorage point, loosely attaching the medial aspect of lower lobes to the mediastinum. The base of the IPL can either merge with the diaphragmatic pleura or end as a free falciform edge, analogous to peritoneal reflections within the abdominal cavity.^2,3^ Early radiological studies on the CT appearance of the inferior pulmonary ligament (Figure 1B–C) reported demonstration of the left IPL in 38–67%, the right IPL in 12–37%, both IPLs in 17–27%, and at least one IPL in 42–78%, however, the emergence of volumetric CT scanning and improved image resolution in subsequent decades has enhanced visualization of these thin anatomic structures.^2,3^ At its lower aspect, the IPL may be contiguous with loose connective tissue within the intersegmental septa (ISS), initially described as thin linear structures between the medial (or anteromedial) and posterior basilar segments of the lower lobes in 78% of normal chest CTs, however, additional septa have been subsequently recognized between other segments within all lobes (Figure 1D).^4,5^ For the sake of anatomic completeness, radiologists should also to be aware of the pericardiophrenic bundle (comprising the phrenic nerve wrapped by parietal pleura) which may be visible as an additional thin line more anteriorly than the IPL (Figure 1E), and the inferior accessory fissure or “Twining’s line” (Figure 1F) separating the right lower lobe medial basilar segment from the remaining basilar segments in 5.6% of individuals.^6,7^

**Figure 2.**
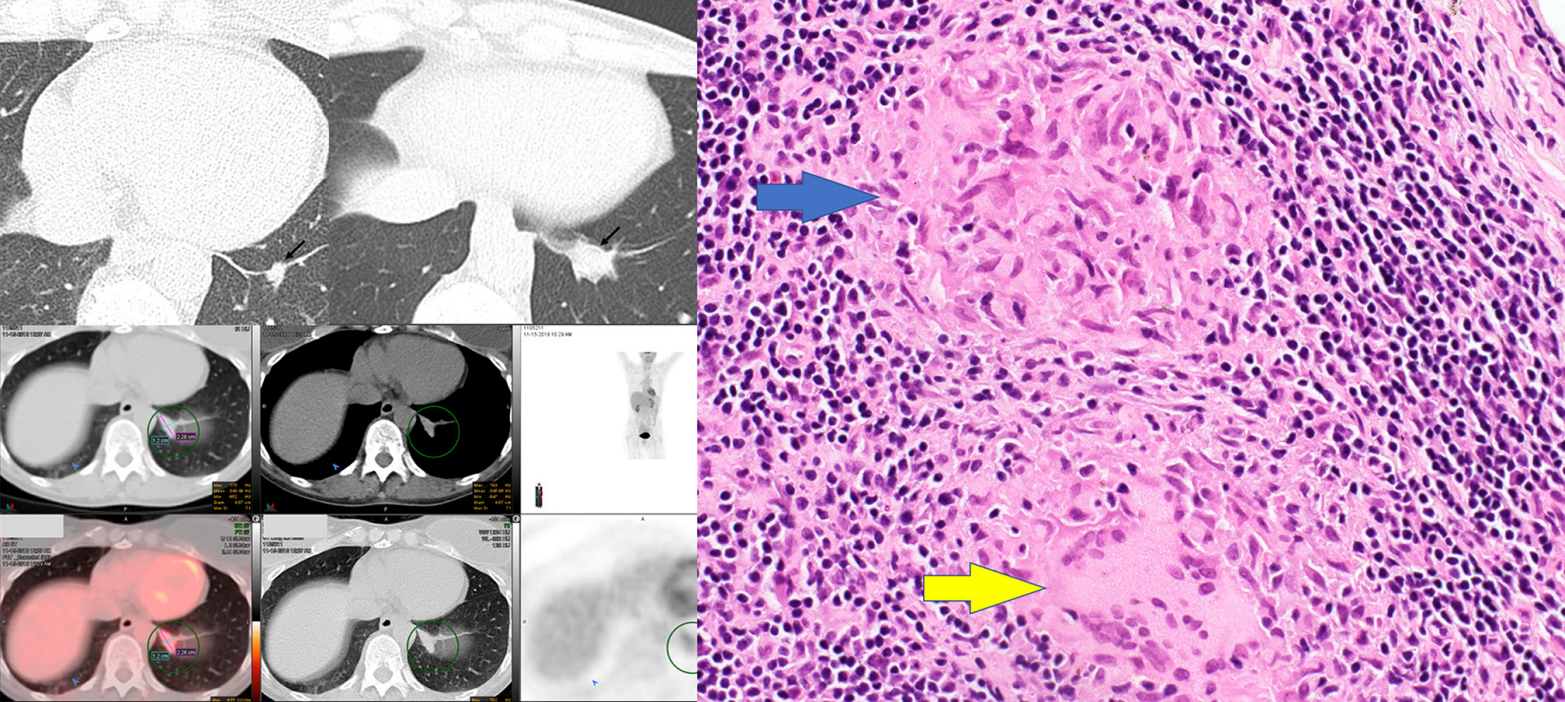
A–D. 57-year-old asymptomatic female with path-proven granulomatous lymphadenitis. 2A: Axial coronary chest CT image on lung windows showing an incidental 14 mm lobulated solid nodule in the left IPL. 2B: Axial chest CT image on lung windows obtained 6 months later demonstrating significant enlargement of the nodule to 2.2 cm. 2C: PET CT of the lesion demonstrating FDG-avidity with SUV of 4.7, interpreted as “concerning for malignancy”. 2D: PATH slide following wedge resection of the lesion. The lymph node contains multiple aggregates of epithelioid macrophages with bent elongated nuclei (blue arrow) and aggregates of multinucleated giant cells (yellow arrow). FDG, fludeoxyglucose; PET, positron emmision tomographyPL, inferior pulmonary ligament; PET, positron emmision tomography.

**Figure 3.**
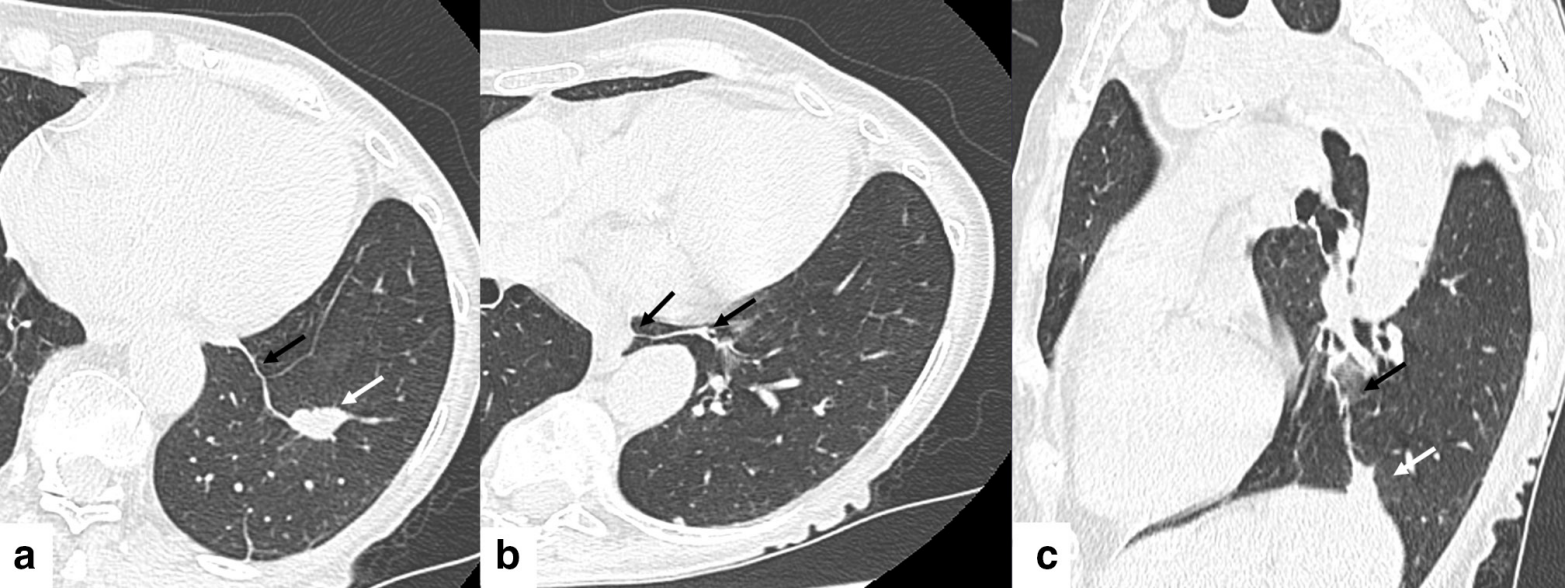
A-C. 75-year-old asymptomatic female with biopsy-proven granulomatous lymphadenitis. 3A: Axial chest CT image on lung windows showing a 2 cm lobulated solid nodule (white arrow) within a septal extension of the left IPL (black arrow). 3B: Axial chest CT image on lung windows at a higher level showing medial extension of the IPL along the left side of the esophagus (black arrows). 3C: Sagital oblique chest CT image on lung windows showing a 2 cm nodule (white arrow) at the base of the IPL, and superior extension of the IPL (black arrow) towards the lower hilar pole.

**Figure 4.**
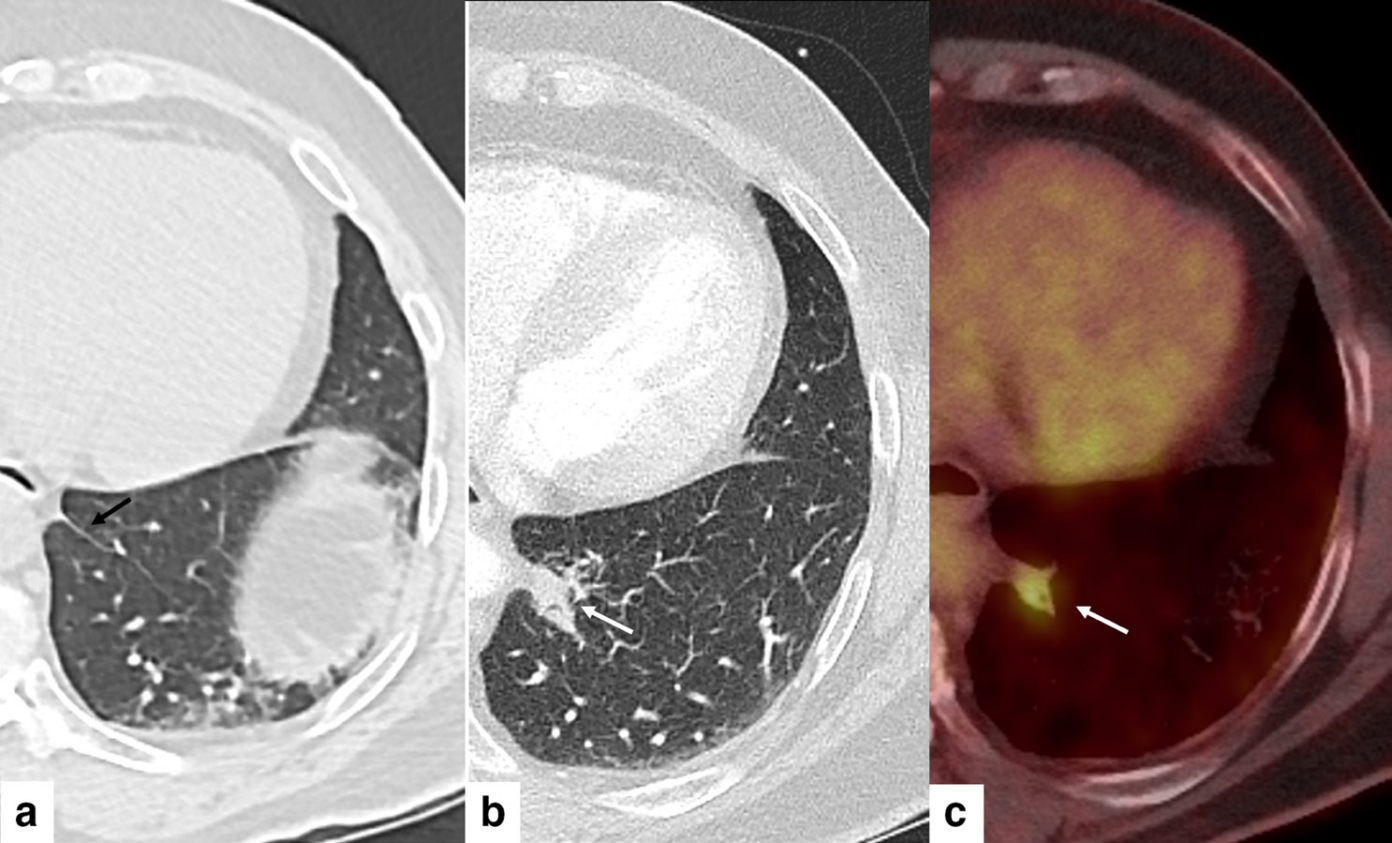
A-C. 65-year-old asymptomatic female with biopsy-proven granulomatous lymphadenitis. 4A: Baseline chest CT on lung windows showing a normal left IPL (black arrow). 4B: Repeat chest CT on lung windows performed 7 months later as part of aortic aneurysm surveillance showing a new lobulated 2.6 cm nodule within the left IPL (white arrow). 4C: PET CT demonstrated FDG-avidity with SUV of 4.6 within the nodule (white arrow), interpreted as “suspicious for malignancy”.

As there are lymphatic channels within these pleural reflections and septa, inflammatory lymph nodes at these sites may mimic a parenchymal nodule. In the authors’ experience, granulomatous lymphadenitis in particular may be the underlying etiology of inflammatory nodes within the IPL and can result in referral to pulmonologists or cardiothoracic surgeons when concern is raised for a malignant lung lesion (Figures 2–4). Granulomatous inflammation within the lung has a broad differential, including infectious (most notably fungal and mycobacterial pathogens) and non-infectious processes (including sarcoidosis, hypersensitivity pneumonitis, and various vasculitides).^8,9^ Granulomatous inflammation is the most common pathologic finding for a benign lung nodule that has undergone resection.^10^ While the majority of necrotizing granulomatous lung lesions are infectious in etiology, 25–39% of granulomatous lung lesions have histopathological features supportive of infection but with no identifiable infectious agent on special stains or conventional infectious screening.^10,11^ The specific etiology of these occult infections is likely to vary by geographic region. In a review of 43 patients who had chest CTs and biopsy-confirmed necrotizing granuloma, Thiesson et al reported positive fungal or acid-fast stains in only 28% of cases—as that study was performed in a North American Valley fungal-endemic area, it would seem likely that a significant proportion were the result of an occult fungal infection.^12^ In Japan, Makuwara et al presented their findings from a series of 28 patients with solitary pulmonary nodules resulting from atypical mycobacterial infection (the majority diagnosed by wedge resection, segmentectomy or lobectomy) and found 39% of cases had mycobacterium avium complex detected by polymerase chain reaction (PCR) while being culture negative, and without generalized findings of chronic non-tuberculous infection on CT.^13^ There is significant overlap in imaging appearances (*e.g.* spiculation and lobulation), fludeoxyglucose (FDG)-avidity, and patient demographics (including age and smoking history) in patients with malignant *vs* granulomatous lung nodules, which renders distinguishing these entities a diagnostic challenge.^12,13^ GL is a recognized and frequently infectious phenomenon with a similarly broad differential diagnosis as granulomatous lung lesions.^14^ GL may affect various nodal groups throughout the body depending on the particular infectious agent, with cervical lymphadenitis from tuberculosis (scrofula) as one of the better known forms.^14^ A literature search reveals no other reports of GL affecting lymph nodes within the IPL, however, this is likely in part related to erroneously ascribing these lesions to parenchymal-based “nodules” in clinical practice, as evidenced by our own experience. It is unclear why lymph nodes within the IPL are susceptible to granulomatous inflammation, however, one could speculate that these nodal groups may be preferentially affected from a gravitational effect related to greater lymphatic clearance within the lower lungs.^15^ Patients may be asymptomatic with lesions detected as an incidental isolated finding and no demonstrable primary sources of inflammation elsewhere.

## Conclusion

An understanding of the anatomy of the IPL and recognition of its chest CT correlate are helpful in distinguishing a lymph node within the ligament from a truly parenchymal-based nodule. In the authors’ experience, a lobulated nodular density within the IPL should prompt consideration of granulomatous lymphadenitis, rather than automatically invoking the range of differential diagnoses that a similar lesion within the lung would ordinarily portend. A more conservative initial management strategy for patients with this imaging constellation may therefore be appropriate, such as a short-term (6–8 weeks) follow-up CT before proceeding to either biopsy or surgical resection.

## Learning points

- Granulomatous lymphadenitis should be included in the differential diagnosis for lobulated nodular lesions within the inferior pulmonary ligament.
- Short-term follow-up imaging can be considered over more invasive management when imaging findings are more typical of granulomatous lymphadenitis than a parenchymal-based lesion.
